# An exploration of how clinician attitudes and beliefs influence the implementation of lifestyle risk factor management in primary healthcare: a grounded theory study

**DOI:** 10.1186/1748-5908-4-66

**Published:** 2009-10-13

**Authors:** Rachel A Laws, Lynn A Kemp, Mark F Harris, Gawaine Powell Davies, Anna M Williams, Rosslyn Eames-Brown

**Affiliations:** 1 Centre for Primary Health Care and Equity, School of Public Health and Community Medicine, University of New South Wales, Sydney NSW 2052, Australia

## Abstract

**Background:**

Despite the effectiveness of brief lifestyle intervention delivered in primary healthcare (PHC), implementation in routine practice remains suboptimal. Beliefs and attitudes have been shown to be associated with risk factor management practices, but little is known about the process by which clinicians' perceptions shape implementation. This study aims to describe a theoretical model to understand how clinicians' perceptions shape the implementation of lifestyle risk factor management in routine practice. The implications of the model for enhancing practices will also be discussed.

**Methods:**

The study analysed data collected as part of a larger feasibility project of risk factor management in three community health teams in New South Wales (NSW), Australia. This included journal notes kept through the implementation of the project, and interviews with 48 participants comprising 23 clinicians (including community nurses, allied health practitioners and an Aboriginal health worker), five managers, and two project officers. Data were analysed using grounded theory principles of open, focused, and theoretical coding and constant comparative techniques to construct a model grounded in the data.

**Results:**

The model suggests that implementation reflects both clinician beliefs about whether they should (commitment) and can (capacity) address lifestyle issues. Commitment represents the priority placed on risk factor management and reflects beliefs about role responsibility congruence, client receptiveness, and the likely impact of intervening. Clinician beliefs about their capacity for risk factor management reflect their views about self-efficacy, role support, and the fit between risk factor management ways of working. The model suggests that clinicians formulate different expectations and intentions about how they will intervene based on these beliefs about commitment and capacity and their philosophical views about appropriate ways to intervene. These expectations then provide a cognitive framework guiding their risk factor management practices. Finally, clinicians' appraisal of the overall benefits versus costs of addressing lifestyle issues acts to positively or negatively reinforce their commitment to implementing these practices.

**Conclusion:**

The model extends previous research by outlining a process by which clinicians' perceptions shape implementation of lifestyle risk factor management in routine practice. This provides new insights to inform the development of effective strategies to improve such practices.

## Background

Lifestyle risk factors such as smoking, poor nutrition, excessive alcohol consumption, and physical inactivity are a major cause of preventable mortality, morbidity, and impaired functioning [1,2]. The World Health Organisation estimates that 80% of cardiovascular disease, 90% of type 2 diabetes, and 30% of all cancers could be prevented if lifestyle risk factors were eliminated [1]. Primary healthcare (PHC) has been recognised as an appropriate setting for individual intervention to reduce behavioural risk factors because of the accessibility, continuity, and comprehensiveness of the care provided [3]. A growing body of evidence suggests that brief lifestyle interventions delivered in PHC are effective [4-8], and the 5A's principle of brief intervention (ask, assess, advise, assist, and arrange) has been widely endorsed in preventive care guidelines [9-12].

Despite this, implementation of risk factor management in routine practice remains low. Screening for lifestyle risk factors does not occur routinely, and only a fraction of 'at risk' patients receive any intervention in PHC [13-16]. Furthermore, studies suggest that when lifestyle intervention is provided it tends to be limited to asking and giving advice on the health risks of the behaviour rather than providing assistance, referral, or follow up needed to support behaviour change [17,18]. The findings of intervention studies aimed at enhancing risk factor management practices have been mixed and often disappointing [19-22]. These studies have used a range of intervention strategies; however, they provide little information about the theoretical or conceptual basis for their choice of intervention and limited contextual data. This suggests that to improve practices a better conceptual understanding of the factors impacting on the implementation of lifestyle risk factor management in routine PHC is required.

Research examining lifestyle risk factor management practices has consisted predominantly of descriptive studies of barriers or enablers, or cross sectional studies of self-reported practices conducted in general practice. These studies have consistently identified the importance of clinician beliefs, including perceptions about role congruence [23-26], self-efficacy [18,27-29], beliefs about effectiveness of interventions [24,25,30-33] and patient motivation [23,34], concern regarding client acceptance [23-25], as well as personal lifestyle behaviours [24,35,36]. Few studies have been conducted beyond general practitioner (GP) PHC providers. Studies among PHC nurses, including community nurses, and registered and licensed practical nurses in USA and Finland, have also reported the importance of clinician beliefs and attitudes, mirroring the findings in general practice [36-39].

Our previous research suggests that those who frequently address risk factors with their patients have different beliefs and attitudes from those who do so less frequently [40]. However, as cross-sectional studies these can provide only limited insight into the way clinician perceptions shape risk factor management practices, and the impact of structural or contextual factors on this. A better conceptual understanding of how clinician beliefs and attitudes influence the implementation of risk factor management in PHC is required to guide the development of effective strategies to improve practice.

This study builds on our previous cross-sectional study [40] and aims to: describe a theoretical model for understanding how clinician perceptions shape their implementation of lifestyle risk factor management in routine practice; and discuss the implications of the model for developing interventions to improve these practices.

## Methods

This study used grounded theory principles, a research method designed to generate a theoretical explanation of a social phenomenon that is derived from (grounded in) empirical data rather than from a preconceived conceptual framework [41], and therefore well suited to understanding process from the perspective of participants [42]. The approach to grounded theory adopted in this study was informed by a constructionist perspective [43] which assumes that neither data nor theories are discovered but constructed based on shared experiences between researchers and participants [43]. Hence, the model produced is a construction of reality offering plausible accounts and explanations rather than verifiable knowledge.

### Study setting and context

This research was part of a larger feasibility project, the details of which have been reported elsewhere [44,45]. In brief, the project aimed to develop and test approaches to integrating the management of lifestyle risk factors into routine care among PHC providers outside of the general practice setting. It involved three community health teams from two Area Health Services (AHS) in the state of New South Wales (NSW), Australia. In NSW, AHS are responsible for providing all hospital- and community-based healthcare apart from general practice and PHC services for specific population groups such as Aboriginal and Torres Strait Islanders. Community health services are the second largest provider of publicly funded PHC services to the general population after GPs [46].

All eight AHS in NSW were invited to express interest in participating in the study and to nominate suitable teams. A total of three community health teams were selected from two of three AHS who expressed interest. Selection was based on the capacity of the team to be involved and the relevance of risk factor management to the type of service provided and healthcare context. Teams were also selected to maximise the variability in team characteristics including provider type, team location (co-located or not), geographical locality, management structures, and health system context.

Team one (n = 35) was a generalist community nursing team with both enrolled and registered generalist community nurses, located in a metropolitan area. Team two (n = 16) was a co-located multi-disciplinary community health team from a rural area, while team three (n = 10) consisted of PHC nurses, Aboriginal health workers, and allied health practitioners providing PHC services to rural and remote communities that generally did not have access to other health services such as a GP (see Additional file 1 for a description of the role of the various community health providers involved in the project). In each of the teams, a baseline needs assessment was conducted to determine current lifestyle risk factor management practices, factors shaping practices, and supports required to improve practices. This needs assessment then informed the development and implementation of a capacity building intervention to enhance practices which was tailored to the needs of each team. Following a six-month implementation period further data was collected to determine changes in practices and factors influencing uptake of practices.

### Data sources and collection procedures

This study utilised two sources of data collected as part of the larger feasibility project: semi-structured interviews with participants prior to and six months following the capacity building intervention undertaken with each team; and project manager journal of reflections and observations recorded throughout the feasibility project.

As part of the feasibility project, semi-structured interviews were conducted with a purposeful sample of participants across the three teams at baseline (n = 29) and six months following the team capacity building intervention (n = 30). At baseline, the aim was to interview a sample of clinicians from across the three teams who varied in profession and role (enrolled and registered nurses, allied health staff, Aboriginal health workers and managers), experience, and geographical location. The same participants were invited to take part in an interview post-intervention (where possible) to provide comparative data on the same individuals over time. A concerted effort was also made to identify and approach to take part in an interview those who felt less positive about the project and risk factor management in general. These clinicians were identified through response on a post-intervention survey and through discussions with managers and project officers responsible for local implementation.

Full details of the data collection procedures for the qualitative interviews have been reported elsewhere [40,45]. In brief, the baseline interviews were conducted by the project manager (lead author RL) and covered issues related to barriers, enablers, and capacity to undertake risk factor management from the perspective of both clinicians and managers (Table 1). Following the project, an evaluation officer (REB) not involved in implementing the team intervention conducted interviews to explore participants' experience of attempting to integrate risk factor management into routine work (Table 1). Interviews at baseline and post-intervention lasted between 20 and 75 minutes, and were tape recorded with participants' permission and transcribed verbatim. The project manager (lead author RL) also kept a journal throughout the two-year project to record reflections and observations following interaction with clinicians and managers during field visits and following participant interviews. All journal notes were typed and included in the analysis for this study.

**Table 1:** Topic guide for baseline and post-intervention interviews conducted as part of the feasibility project

**Baseline interviews**	**Post-intervention Interviews**
• Overview of job role	• General impressions of the project
• How addressing SNAP risk factors fits with the job role^1^/core business of team or service^2^	• Case example--last client with a risk factor^1^
• Approach to addressing SNAP risk factors (client case example)^1^	• Feasibility of risk factor screening/intervention
• Work priority to address SNAP risk factors^1^	• Barriers/enablers risk factor screening/intervention
• Confidence to address SNAP risk factors^1^	• Case example--comfortable to address^1^
• Barriers and enablers to addressing SNAP risk factors in routine work	• Case example--not comfortable to address^1^
• Support and resources required to address SNAP risk factors in routine work^1^/strengthen team capacity to address risk factors ^2^	• Perceived effectiveness of intervening^1^
• Opinion on strength of local referral networks and programs to support risk factor management^2^	• Congruence with core business of the team and organisation^3^
• Opinion on team climate and any competing priorities in implementing the project^2^	• Process of project implementation (degree of consultation and model adaptation to suit team)^3^
	• Change in approach to addressing SNAP risk factors
	• Views about continuation of risk factor management as part of professional role^1^/team or service^3^
	• Support required for continuation of risk factor management practices in professional role^1^/team or service^3^
	• Project benefits (personal and professional^1^/team or service^3^)

SNAP: Smoking, nutrition, alcohol and physical activity

^1^Team and service managers only

^2^Team/service managers and project officers only

### Data analysis and model development

Developing the model involved two main stages of analysis. First, a preliminary model was developed by analysing a purposeful selection of baseline interviews (n = 18) of participants who also participated in an interview following the project, allowing for comparison over time. Analysis at this stage involved open and focused coding to identify key theoretical categories and ideas about how these were related [47]. From this process, a preliminary model was constructed and compared to relevant theories in the literature in order to identify 'conceptual gaps', heightening the researcher's theoretical sensitivity [48,49].

The second stage of analysis involved refining the preliminary model through analysis of additional interviews (n = 30) and the project managers' journal notes. In line with grounded theory principles [41,50], 10 interviews were theoretically sampled from the existing interview dataset. A sampling frame was devised (Table 2) in order to identify those with a diverse range of attitudes and practices relevant to the evolving model. Clinician response on a risk factor management survey undertaken at baseline and post-intervention was used to identify clinicians meeting the sampling criteria (details of the survey reported elsewhere [40]). A further 20 interviews were purposefully selected including post-intervention interviews for those who had participated in an interview at baseline (n = 18) and interviews with project officers (n = 2) involved in implementing the project locally. Analysis at this stage involved assessing how well the focused codes developed in the preliminary model fitted the new data. This process resulted in the revision of some categories (for example, to include additional properties and dimensions) and the development of additional categories to reflect the data. Baseline data was then recoded using the new and revised categories to ensure the conceptual fit with the data. Theoretical coding was then used to specify the possible relationships between the categories developed during focused coding to construct a coherent analytical story [41,42,47]. Preliminary ideas about relationships were tested by going back to the data in accordance with grounded theory principles of moving between induction and deduction in the development of theory [42].

**Table 2:** Criteria used to theoretically sample interviews to include in the analysis

**Factors related to key categories in the baseline model**
Clinicians who scored low^1 ^or high^2 ^on the following attitude items completed as part of a survey at baseline and/or post-intervention:
• The acceptability of raising risk factor issues with clients
• Perceived work priority
• Perceived effectiveness of addressing lifestyle issues
• Confidence in assessing and managing lifestyle risk factors
• Confidence in applying behaviour change
• Perceived accessibility of support services

Other criteria included
• Clinician types not included in the baseline analysis
• Clinicians reporting change^3 ^in confidence and/or attitudes from baseline to post-intervention:
• Clinicians and managers who have recently joined the team (last six months)

**Clinician screening and intervention practices**
• Clinicians who had low or high levels of self reported screening for lifestyle risk factors at baseline and/or post-intervention^4^
• Clinicians who had low or high levels of self reported intervention for lifestyle risk factors at baseline and/or post-intervention^5^
• Clinicians reporting a change^3 ^in screening and or intervention practices from baseline to post-intervention

^1^Low defined as scores in the clinician risk factor survey in the lowest quartile for those participating in an interview

^2^High defined as scores in the clinician risk factor survey in the highest quartile for those participating in an interview

^3^Change defined as scores increasing from lowest to highest quartile or highest to lowest quartile (baseline to post-intervention)

^4^High screening practices = mean screening score (across risk factors) in the highest quartile for those participating in an interview, low screening practices = mean screening score in the lowest quartile for those participating in an interview

^5 ^High intervener = high frequency of intervention for three or more risk factors and/or high intensity intervention (across risk factors), low intensity intervener = low frequency of intervention for three or more risk factors and/or low intensity intervention (across risk factors).

Throughout the analysis process, constant comparative techniques were used to assist in uncovering the properties and dimensions of each category. This involved comparing data within the same coding group, making comparisons between different clinicians and between the same clinician over time. In line with Strauss and Corbin's [42] notion of axial coding, attention was paid to identifying and comparing the conditions giving rise to an issue, the context in which it was embedded, the strategies used by clinicians to manage this, and the consequences for clinicians beliefs and practices. Insights gained were recorded in the form of memos throughout the analysis process. NVivo 7.0 software [51] was used to attach codes to text, record memos, and diagrams, as well as facilitate the retrieval of data.

One member of the research team (RL) undertook the analysis. To avoid the researchers' views being 'imposed' on the data, RL documented assumptions prior to analysis and kept an audit trail to document coding decisions, which included extensive use of participant quotes to justify the approach taken [52]. A conscious decision was made not to use member checking, a process of cross-checking findings and conclusions with participants. As the purpose of the analysis was to code all responses and organise into a new higher order theoretical model, it was not expected that participants would be able to recognise their individual contributions or concerns. It was therefore not appropriate to seek 'validation' from individual participants. Instead, a number of other techniques were used to ensure interpretations were grounded in the data. These included the use of constant comparisons, memo writing, extensive use of participant quotes, and discussing coding frameworks and preliminary theoretical ideas with two other members of the research team (MH and LK) for the purpose of gaining other perspectives and challenging assumptions rather than to reach agreement.

### Ethics

The project was approved by the UNSW Human Research Ethics Committee (HREC) and the HREC in each AHS.

## Results

The final sample in this study included 48 interviews with 23 clinicians, three team managers, two senior community health managers, and two project officers. Fourteen clinicians and four managers were interviewed twice, at the beginning and end of the project. The sample included generalist community nurses, child and family nurses, a range of allied health providers, and one Aboriginal health worker. All were female, with a wide range of professional experience. The interview sample included in this study was broadly representative of clinicians from the three teams (Table 3). However, allied health practitioners and child and family nurses from team two were over-represented and males under-represented in the interview sample. This reflected the purposeful and theoretical sample techniques that aimed to include a diverse range of clinician types and those with varying levels of attitudes and practices related to the management of lifestyle risk factors.

**Table 3:** Characteristics of clinicians included in the interview sample compared to all clinicians

	**Clinician interviews included in analysis (n = 23)**	**All clinicians**^1^ **(n = 61)**
**Age Category**	No. (%)	No. (%), n = 57
18 to 24 years	2 (8.7)	2 (3.5)
25 to 34 years	2 (8.7)	6 (10.5)
35 to 44 years	8 (34.8)	16 (28.1)
45 to 54 years	8 (34.8)	26 (45.6)
55 to 64 years	3 (13.0)	7 (12.3)
		
**Clinician experience**	**Mean (std), range**	**Mean (std), range n = 60**
Years in profession	21.0 (11.4), 1-35.0	21.6 (11.0), 1-46.0
Years in community health	8.4 (8.1), 0.5-30.0	10.5 (7.8), 0.5-30.0
Years in team	6.8 (6.5), 0.5-20.0	6.5 (6.1), 0.5-22
		
**Gender**	**No. (%)**	**No. (%), n = 60**
Male	0 (0.0)	3 (5.0)
Female	23 (100.0)	57 (95.0)
		
**Employment**	**No. (%)**	**No. (%), n = 55**
Part time	12 (52.2)	26 (47.3)
Full time	11 (47.8)	29 (52.7)
		
**Clinician type**	**No. (%)**	**No. (%), n = 60**
Generalist community nurse (registered nurse)	12 (52.2)	37 (61.7)
Generalist community nurse (enrolled nurse)	3 (13.0)	11 (18.3)
Child and family nurse	2 (8.7)	2 (3.3)
Allied health practitioner	5 (21.7)	8 (13.3)
Aboriginal health worker	1 (4.3)	2 (3.3)
		
**Team**	**No. (%)**	**No. (%), n = 61**
Team one	9 (39.1)	35 (57.4)
Team two	10 (43.5)	16 (26.2)
Team three	4 (17.4)	10 (16.4)

^1^Demographic information collected at baseline as part of clinician survey. Missing data: age n = 4; gender n = 1, employment n = 6, clinician type n = 1.

### Model Overview

**Figure 1:**
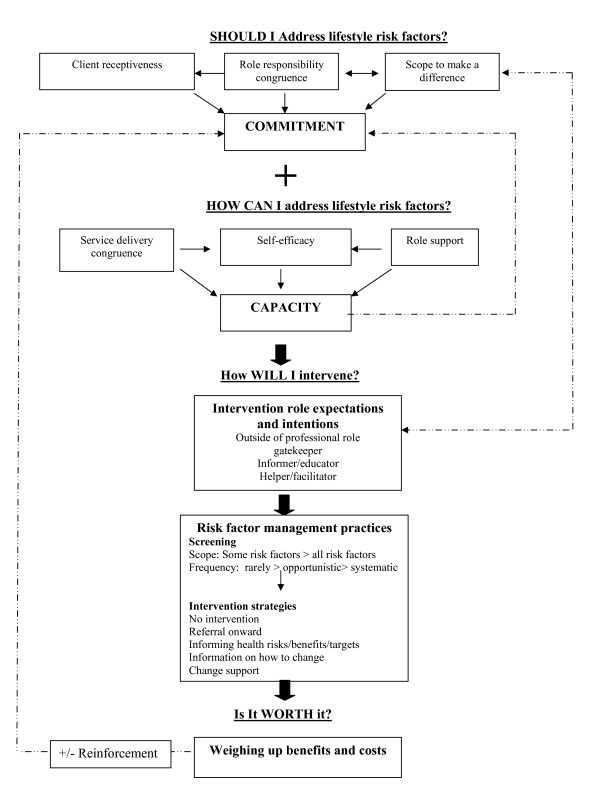
**The practice justification process: A model of how clinician perceptions shape their risk factor management practices**.

The theoretical model is shown in Figure 1. It suggests that clinician perceptions shape their risk factor management practices through the process of 'practice justification'. This involves justifying risk factor management practices as a legitimate, 'doable,' and worthwhile component of the role. This process consists of five main interrelated factors:

1. Developing commitment (Should I address lifestyle issues?)

2. Assessing capacity (How can I address lifestyle issues?)

3. Formulating intervention role expectations/intentions (How will I intervene?)

4. Implementing risk factor management practices

5. Weighing up benefits and costs of practice (Is it worth it?)

Each of these steps in the model is described below.

### Developing commitment--Should I address lifestyle issues?

First, 'commitment' represents the priority or importance placed on risk factor management in the role, influencing 'if and when' clinicians address lifestyle issues, the amount of time they are willing to invest, and the scope of their practice (type of risk factors addressed and frequency in which this occurred). Commitment in turn appeared to be shaped by three main factors, as outlined in Figure 1: role responsibility congruence, perceptions of client receptiveness, and beliefs about the 'scope to make a difference'.

Clinicians expressed a diversity of views about how addressing lifestyle issues fitted with their role responsibilities. For some, it was simply an assessment task to 'tick off' before getting on with the job of looking after the clients; for others the relevance varied, depending on the clients presenting problem. In contrast, other clinicians saw risk factor management as an integral component of their role in providing holistic PHC, as articulated by this clinician:

'My approach is...holistic health and wellness...so ultimately what I'm looking for is information to assist people being totally healthy and well...So continuing to assess and support lifestyle changes, yeah I do believe it should continue to be part of our role.' (Clinician 23)

Overall, the broader clinicians' perspective of the relevance of lifestyle issues to their role, the more they were willing to invest time in addressing them. These views tended to reflect the model of service delivery adopted by the team/service in which they worked and clinicians' discipline and training. For example, only generalist community nurses or PHC nurses considered addressing lifestyle issues as part of their role in providing holistic care, while the relevance for allied health practitioners depended on the link between risk factor issues and the clients presenting problem. For those with a counselling role (such as psychologist and social workers), screening for lifestyle issues was considered to be in conflict with their client-centered approach, and they considered it only appropriate to address risk factor issues opportunistically when relevant to the clients concerns:

'I think for the nurses, it's very feasible because the nurses tend to be holistic and cover absolutely everything, and I think for the allied health, its still quite feasible, perhaps not all the [risk] factors like the nurses...for the...counselling type people, I think it's been harder for them to do it just because they have such a 'let the client take the direction focus'. (Project Officer 2)

In addition to clinicians' intrinsic sense of their professional responsibility to address lifestyle issues, their perception of client receptiveness was an important driver of their commitment to broach these topics. Clinicians who reported that clients were receptive to them asking about lifestyle risk factors expressed confidence and commitment to raising these issues. Lifestyle risk factors were considered easier to raise, and clients most receptive, when the client was being seen for a preventive or PHC issue, and when the clinician had ongoing contact with the client in a case management role. Some clinicians considered lifestyle issues more difficult to raise when seeing clients in their own home due to clients control over the care agenda and the clinicians assumed role of a 'guest' who does not want to offend their 'host'. Clinicians also deemed clients to be less receptive when they had other pressing problems, or when they were unreceptive to the care process in general. When clinicians expressed concern about client receptiveness, they discussed feeling less confident and committed to broaching lifestyle topics because of the implications a negative reaction might have for their own safety and/or their relationship with the client, as illustrated in this quote:

'You go in there as a single nurse on your own, and if you don't approach the subjects in the right way, you could end up in a little bit of an uncomfortable situation....' (Clinician 18)

Finally, clinician commitment not only reflected their beliefs about their professional responsibility and client receptiveness, but the extent to which they believed that intervening could have a positive impact (labelled 'scope to make a difference': Figure 1). Clinicians were doubtful and sometimes openly pessimistic about whether intervening would make a difference when they:

1. considered the benefits of intervening only at the individual level and in terms of primary prevention of disease;

2. did not see a role for themselves in motivating clients to change behaviour: hence a lack of client motivation was considered a major barrier in certain groups of clients (*e.g*., older clients, those with other pressing problems);

3. judged the effectiveness of intervention in terms of the number of clients achieving the desired behavioural targets;

4. attended clients for one off, or short term services where there was limited opportunity to build rapport or follow up outcomes achieved.

In these circumstances addressing lifestyle issues was considered to be of limited use and hence, commitment to doing this was low, as argued by this clinician:

'If, unfortunately intellectually they can't...take those issues on board, really nothing much you say can alter lifestyle patterns that are from birth. So, I tend...look at what I can change and try to change it, and if I don't think I can, then I just move around it.' (Clinician 4)

In contrast, clinicians were more likely to identify greater scope to make a difference when they:

1. took a broader view of the benefits of intervening beyond the individual, and for the purposes of primary through to tertiary prevention and maintenance of quality of life;

2. viewed their role as facilitators of change: hence a lack of client motivation was not considered a deterrent but part of the process;

3. judged the effectiveness of their intervention in terms of the process of change rather than achieving behavioural targets, considering their intervention as one of many which may impact on the prevalence of lifestyle risk factors at the population level.

Not surprisingly, these clinicians also adopted a broader view of their role responsibilities beyond the presenting issue to providing PHC services to families and communities. Clinicians' belief about their own ability and capacity to effect change was also important in shaping their perceptions about the likely impact of intervening, as discussed below.

### Assessing capacity--How can I address lifestyle issues?

Clinicians' risk factor management practices not only reflected their beliefs about whether they 'should' address lifestyle issues (commitment) but also their beliefs about how they 'can' address lifestyle issues (capacity). Three main components of capacity were identified to be important in shaping practices (Figure 1): self-efficacy, role support, and service delivery congruence.

First, in order for clinicians to feel confident addressing lifestyle issues, they needed to believe that they had the ability to do so, based on internal factors, such as knowledge, skills, experience, and their own lifestyle habits. This has been labelled 'self-efficacy' in the model and appeared to be important in determining the type of intervention offered, as discussed by this clinician:

'...I suppose maybe it's based on how comfortable...or personally confident I feel about offering anything... I certainly would refer to the relevant person but not deal with it specifically myself.' (Clinician 6)

To feel confident offering an intervention themselves, clinicians discussed the importance of having an understanding of various intervention strategies either through their own experience of lifestyle change or through their work with clients. However, they also recognised a need for a sound grasp of behaviour change skills, such as motivational interviewing, if they were to move beyond providing information and advice to facilitating behaviour change

Perceptions about capacity not only reflected clinicians' confidence about their own abilities but also external factors such as access to support mechanisms, labelled 'role support' (Figure 1). This included decision support tools (such as screening tools), ongoing training, client education materials, collegial support, and access to referral services for clients. These mechanisms appeared to increase clinicians' confidence to intervene by enhancing perceptions of self-efficacy, and by providing 'back up' support and 'something tangible' to offer clients:

'Now they have somewhere they can refer them to because before [the project] even if they wanted to address it, it was like, 'oh well, what's the point, where can I refer them to'... but now that they know that there is actually something, I think it makes a big difference.' (Manager 5)

Data analysis suggests that access to these support mechanisms is dependent on having wider system level support for risk factor management at the service and organisational level, including good linkages with support services.

Finally, the work environment was important in shaping perceptions about capacity, in particular the fit between risk factor management and ways of working (labelled 'service delivery congruence': Figure 1). As part of the project, teams were consulted about the most appropriate way for them to address lifestyle issues, given their current way of working. This consultation process was identified as an important moderator to developing approaches that fitted with the mechanics of everyday practice. At the macro-level, the extent to which risk factor management was seen to fit with the model of service delivery was also important in shaping clinician's beliefs about the opportunities they had for implementation. For example, all community nurses interviewed in team one identified the focus on providing post-acute care as limiting the time available for health promotion activities. Some allied health providers also questioned their capacity to address lifestyle issues peripheral to the reason for referral, given that they were solo practitioners with long waiting lists and limited ongoing contact with clients. In contrast, team three considered risk factor management as central to delivering PHC services to rural and remote communities with a focus on early intervention and prevention, as summed up by this participant:

'...we have chronic disease prevention and early intervention as one of the five priority health areas...so it [risk factor management] fits really well into...our core business.' (Team 3)

### Formulating intervention role expectations/intentions--How will I intervene?

Analysis of the data suggests that clinicians formulate different expectations and intentions about how they will intervene based on their beliefs about commitment and capacity and their philosophical views about appropriate ways to intervene (Table 4, Figure 1). Philosophical views appeared to reflect a diversity of beliefs about the determinants of lifestyle behaviours and how they should be best managed. Role expectations ranged from seeing lifestyle risk factor management as completely outside of the professional role and best managed through population health approaches, to those who considered they had an important role to play in facilitating behaviour change by providing tailored support strategies (Table 4). These role expectations and intentions appeared to act as a cognitive framework or mindset shaping clinicians' intervention practices.

**Table 4:** Intervention role expectations and intentions: Description and illustrative quotes

**Intervention role expectations/intentions**	**Philosophical views about appropriate ways to intervene**	**Illustrative quotes**
**Expectations--Outside of Professional Role** Intervention considered outside of the professional role, best addressed through population health approaches Intentions: Do not intervene to address lifestyle issues	**Population Health Perspective**: Lifestyle behaviours best tackled through addressing underlying determinants of risk taking behaviour	'It wouldn't be us that would be able to take that extra work on...It'd have to be like those ones that do the programs like population [health]Like you people and all that that get funded for these things would have to carry it further.' (Clinician 22)

**Expectations--Gatekeeper** Intervention considered outside of scope of professional expertise and job role, best addressed by qualified experts. **Intentions**: Refer clients onwards to qualified experts/specialist service	**Medical perspective**: Lifestyle behaviours are complex and require specialist input from qualified experts	It's not my job to get people to quit smokingIf they want to quit smoking I would give them the quit line numberI don't have...those skills...if I was a drug and alcohol worker it'd be a different story, but I'm not.' (Clinician 15)

**Expectations--Informer and educator** Ensure client has sufficient information to make an informed choice about lifestyle behaviour. Can only provide intervention to those willing to change. **Intentions** Provide information on health risks/benefits of lifestyle risk factors to all clients. Provide additional assistance to motivated clients.	**Individual perspective **(individual autonomy and self empowerment): Lifestyle behaviours are personal choices that people make and as such should be respected. Individuals need to take responsibility for change	'I really leave it up to them--it's their decision what they're going to do, but at least I can give them the information so they can reach a decision whether to keep on smoking or stop.' (Clinician 7)

**Expectations- Helper or facilitator** Help move clients towards change over time by acting as a facilitator. Synergistic role with other providers and population health approaches **Intentions**: Facilitate clients to change their behaviour through providing tailored support strategies.	**Socio-ecological perspective** Lifestyle habits are complex behaviours influenced by a range of social and environmental factors. Multiple interventions required at individual and population level to effect change.	'If everybody got together and said these risk factors well then people are going to think ...and obviously it's working with the...TV advertising...our smoking rates are going down....' (Clinician 14)

### Risk factor management practices

Clinicians' risk factor management practices varied according to the approach adopted for assessing lifestyle risk factors (opportunistic versus systematic), the type of risk factors addressed (all or selective risk factors), and the range of intervention strategies used (Figure 1). Practices varied between clinicians and also by the risk factor being addressed (for some clinicians). These variations can be best understood in terms of the key model categories of commitment, capacity, and intervention role expectations and intentions.

A small number of clinicians reported infrequently broaching lifestyle issues. This reflected both a lack of commitment and capacity. First, lifestyle risk factors were not generally considered relevant to the clients presenting problem, and thus clients were unlikely to be receptive to discussing these issues. Screening for lifestyle risk factors was also not part of their usual work process, they reported having limited opportunities to intervene and they lacked the necessary knowledge, skills, and access to support tools/resources. Clinicians who reported adopting an opportunistic approach to asking about selective risk factors with particular clients did not routinely ask about lifestyle issues as part of existing work processes. Hence, they took an opportunistic approach to broaching these topics when the lifestyle issues were considered relevant to the clients presenting problems, and when the client was likely to be interested and able to make lifestyle changes. In contrast, those clinicians who reported using a systematic approach to asking about most lifestyle issues with the majority of their clients took a broader view of the relevance of lifestyle risk factors to their role and/or asking about lifestyle issues was integrated into the standard assessment process.

Once risk factors were identified, clinicians' intervention practices ranged from providing no intervention (one clinician) to providing personalised support for lifestyle change tailored to the clients' situation (Figure 1, Table 5). Intervention strategies differed in terms of the time, knowledge, and skill required to deliver them. For example, referring clients onward to more specialist service was a one off task requiring minimal skill and investment of time. In contrast, providing personalised support for lifestyle change required skills in behaviour change counselling and more time to engage clients in the change process that often occurred over a number of consultations. The choice of intervention strategies used largely reflected clinicians' intervention role expectations and intentions, as discussed in the previous section.

**Table 5:** Intervention strategies: Illustrative quotes

**No intervention**
'I would never discuss the interventions. We never got that far...we do not have clients that these things are practical for.' (Clinician 22)
**Referral onward**
'I think the most I have done is referred someone to Quitline but in terms...of doing anything I haven't really done a lot.' (Clinician 5)

**Informing of health risks/benefits and lifestyle targets**
'Recently, I saw a gentleman...probably early 70s who has obviously been a smoker all his life. He had quite a nasty area on his wound that was probably going to take quite a while to heal. I could just present him with the factors that I knew about smoking, and encourage him probably to reduce that intake, that we all know.' (Clinician 18)

**Providing information or advice on how to change**
'I asked him...if it was time that he thought he could probably give up smoking, that this was... impairing his breathing...and I pointed out to him that I could probably help him, refer him to a quit smoking campaign and he said he would like to be able to stop smoking but he can't so...I just left it with him...and if he felt that he needed, he wanted to pursue it then I could point him in the right direction to do that. That's all I can do in that situation....' (Clinician 20)

**Change support**
'The client last week said she'll cut down on her drinking. She is pregnant....She only drinks six cans of bourbon and coke a day now, probably half what she'd normally. we try and build up a little helping network around them and try and sort out why they are acting like that, we need to help them change their living environments or think that there is help to do it' (Clinician 10)

### Weighing up the benefits and costs--Is it worth it?

Finally, clinicians' appraisal of the overall benefits versus costs of their risk factor management practices acted to positively or negatively reinforce their commitment to addressing lifestyle issues (Figure 1). Some clinicians expressed uncertainty about whether addressing risk factors was a worthwhile component of their role because of their limited capacity for implementation (labelled role insufficiency), suggesting that perhaps this should be taken on by others. Other clinicians argued that the costs in terms of time and potential client resistance were not justified, given the limited perceived benefits in their client group. These clinicians expressed resentment that risk factor screening was a requirement of the service (labelled 'role resistance'), as illustrated in this quote:

'There have been no benefits [of the project] but extra work...At least half hour, if not an hour of extra work...Per client...with a negative result.' (Clinician 22)

In contrast, others endorsed risk factor management as a worthwhile practice due to the potential benefits of intervening and their capacity for implementation, resulting in professional satisfaction (labelled role verification), as summed up by this clinician:

'It hasn't been difficult to incorporate... I think in fact it's quite good to have some salient points to hit upon and it really hasn't made the assessment process that much unduly long...I think it's a very positive thing.it's what community health is all about.' (Clinician 4)

## Discussion

The theoretical model presented in this study extends previous descriptive and cross-sectional studies by providing insight into the process by which clinician beliefs and attitudes shape the implementation of risk factor management in routine PHC practice. Given the many competing demands facing PHC clinicians and their inability to address all preventive care needs [53,54], the findings suggest that clinicians rationalise their approach to managing lifestyle risk factors. This involves making judgements about the extent to which addressing lifestyle issues is considered a legitimate, doable, and worthwhile component of the role. The model suggests that implementation reflects both clinician beliefs about whether they should (commitment) and can (capacity) address lifestyle issues, and these beliefs are shaped by a range of patient, provider, and contextual factors. Beliefs about commitment and capacity, together with moral views about appropriate ways to intervene, all shape clinicians intentions about how they will intervene. This then provides a cognitive framework guiding their risk factor management practices. Finally, clinicians appraisal of the overall benefits and costs of addressing lifestyle issues acts to positively or negatively reinforce their commitment to implementing these practices.

The model constructs are largely in line with previous quantitative and qualitative studies suggesting that a combination of patient, contextual, and provider factors shape clinicians management of lifestyle risk factors. For example, previous studies have found that higher risk patients [14,32,55-57], those perceived to be more motivated [58], and the least disadvantaged [55,59,60] are more likely to receive lifestyle intervention In line with our findings, contextual factors related to the service delivery environment have also been found to influence practices in previous studies including the length and number of consultations [55,61], provider workload [62], and purpose of the visit [32,59,60]. Similarly, access to role support, such as training [18,55,63], decision support tools [32,36,55,63,64], collegial support [58,65], and client education materials [66-68] have all been associated with provision of lifestyle intervention.

Our findings offer fresh insights by suggesting that these patient and contextual factors shape practice through their influence on providers' beliefs and attitudes. For example, clinicians are more committed to providing intervention to patients considered to be highly motivated because they perceive that these patients will be receptive, and the scope to make a difference is high. Similarly, access to role support and the service delivery context all influence perceptions about capacity. The model also highlights the synergistic relationship between commitment and capacity. Clinicians who perceive that they have the capacity to address lifestyle issues are more likely to believe that intervening will have a positive impact (scope to make a difference) reinforcing their commitment. Clinician commitment appears to be a prerequisite for capacity-building interventions to be effective. Finally, the model is unique in suggesting that beliefs about commitment and capacity, together with moral views about appropriate ways to intervene, all shape clinicians intentions about how they will intervene, which in turn determines the type of intervention strategies used.

At a theoretical level, the model has much in common with a model developed by Shaw and colleagues in the management of alcohol and other drugs (AOD) [69]. Shaw's model suggests that role perceptions, in particular role legitimacy (perceived boundaries of professional responsibility and right to intervene) and role adequacy (self-efficacy) form the foundation for health professionals motivation and satisfaction to respond to AOD issues [69,70]. Role support (help and advice from colleagues, supervisors, and other organisations), AOD education, and work experience were in turn thought to influence these role perceptions [69]. However, the current model extends Shaw's model in a number of ways. Firstly, it suggests that beliefs about outcomes, in particular beliefs about the 'scope to make a difference' and appraisal of benefits versus costs, are important in shaping commitment/motivation. It also expands the concept of role adequacy beyond self-efficacy to also include the extent which addressing lifestyle issues fits with current ways of working (self-delivery congruence). It suggests that role perceptions shape practice through intentions/expectations that also reflect philosophical views about appropriate ways to intervene.

The constructs identified in the model are largely in line with the main theoretical domains suggested by dominant psychological theories of motivation and action [71-76], including beliefs about capabilities, beliefs about consequences, and normative beliefs. Research suggests that these domains also apply to health professional practice, explaining on average 31% of 59% of the variance in clinician behaviour and intentions respectively [77]. These theories focus predominantly on individual cognitive factors shaping behaviour and do not explicitly include contextual/organisational factors and role beliefs. This is not surprising, given that these theories were developed to understand individual health behaviours rather than clinical practices. In contrast, the study model explicitly identifies the importance of the service delivery environment, as well as role beliefs--in particular beliefs about role congruence, role support, and intervention role expectations and intentions--in shaping risk factor management practices. As a result, the model provides new insights into theoretical constructs likely to be important in understanding the management of lifestyle risk factors that may apply more broadly to other clinician behaviours.

The study findings point to a number of possible leverage points for interventions to improve the lifestyle risk factor management practices of PHC clinicians. First, consideration should be given to tailoring the approach to lifestyle screening and intervention to suit the commitment and capacity of various healthcare providers. The findings suggest that it may be unrealistic to expect most providers to undertake all steps recommended in the widely endorsed 5A's approach to brief intervention [10,12]. A minimal approach to intervention would be to refer clients requiring intervention onwards to support services (arrange). This approach requires a minimal investment of time, and may best suited to clinicians for whom lifestyle issues are a peripheral component of their role and care is focused on treating a specific problem. The next level of intervention may be to provide brief advice regarding lifestyle recommendations followed by referral (advise and arrange). More intensive interventions, such as providing personalised support for lifestyle change tailored to the clients readiness to change with ongoing follow up and/or referral (advice, assist and arrange), is probably best suited to clinicians for whom lifestyle intervention is central to their role, the model of care is focused on early intervention/prevention, and they have specific knowledge and skills related to behaviour change interventions. At a system level, this tailored approach may be more realistic and facilitate uptake of practices and overall reach of lifestyle intervention to individuals. There is also evidence that minimal approaches (such as asking or brief advice) provided to individuals by more than one health professional can be effective in promoting behaviour change [78].

Second, improving practices is likely to require a range of professional development activities focusing on building positive clinician attitudes, skills, and self-efficacy. In particular, developing skills in behaviour change counselling, such as motivational interviewing approaches, is likely to be important in reducing client resistance and creating positive and effective interactions with clients compared to didactic approaches [79]. The findings suggest that shifting clinicians' views about the value and impact of lifestyle intervention is critical to enhancing commitment. This is likely to require a fundamental shift from a predominantly medical worldview, one that values high-tech interventions and dramatic outcomes, to a behavioural worldview that employs low-tech interventions (talking to people) to achieve small incremental changes in behaviour over time [80]. Given that professional values and norms are transmitted through early professional training, core competencies for lifestyle risk factor management should be integrated into undergraduate and postgraduate training for PHC providers. For existing providers, the inclusion of lifestyle risk factor management into continuing professional development activities will require a systematic and coordinated approach at the service/organisational level.

Third, at a service and organisational level, our findings highlight the importance of the model of service delivery and access to role support. In particular, service delivery models that involve case management, offer continuity of care, and focus on early intervention and prevention are likely to be more conducive to implementing risk factor management. This is likely to require policies to support service delivery redesign to improve the balance of preventive care and illness management. Embedding risk factor management activities into existing routines and work processes is also likely to be important along with wider organisational commitment required to sustain role supports, such as access to ongoing training, referral services, and decision support tools. Critical to achieving this will be a supportive policy environment for preventive care in PHC settings. These recommendations are consistent with the elements of the chronic care model that has recently been applied to health risk behaviours [81].

The study has a number of limitations. The model was developed in one PHC setting (state-funded community health services in NSW, Australia) based on the experiences of a limited number of participants and teams. Teams were selected to participate in the feasibility study based on an expression of interest, and hence may have been more interested and motivated to address lifestyle risk factors compared to other teams. Furthermore, participants who agreed to be interviewed may be more engaged in addressing lifestyle issues than participants who declined to be interviewed. However, a range of participants took part in the interviews, including those who felt positive and negative about the project. Furthermore, purposeful and theoretical sampling techniques ensured a wide range of participant were included in the analysis, including clinicians with both high and low levels of practice and a range of different types of practitioners (allied health professionals, registered and enrolled nurses, although all were female) from across the three teams. Insights were also gained from a number of other perspectives, including project officers involved in implementing the capacity building intervention, team managers, senior managers, and observations and reflections obtained by the researcher through prolonged engagement with the teams. Despite this, uncertainty remains about the extent to which model might apply to other PHC settings, such as general practice and other health professional behaviours. Further research is required to assess the usefulness of the model in other settings and contexts. In keeping with a constructivist approach, it is acknowledged that the model has been constructed based on the shared experiences between researchers and participants, and it aims to offer insights and understanding rather than verifiable knowledge.

## Conclusion

The theoretical model presented in this paper suggests that clinician beliefs and attitudes shape the implementation of lifestyle risk factor management through the process of 'practice justification'. This involves justifying risk factor management practices as a legitimate, 'doable', and worthwhile component of the role. The model offers new theoretical insights by suggested the importance of the service delivery environment and role beliefs in shaping practices in addition to individual cognitive factors suggested by psychological theories of motivation and action. Improving practices will not only require a range of professional development activities to build positive clinician attitudes and skills, but attention should be paid to creating models of service delivery conducive to preventive care and providing ongoing role support. Finally, consideration should be given to tailoring the approach to lifestyle screening and intervention to suit the commitment and capacity of various healthcare providers to maximise the reach of lifestyle screening and intervention at the population level. Further research is required to test the model, in particular its application in other settings, and to develop and test the effectiveness of strategies for improving the management of lifestyle risk factors in PHC.

## Competing interests

The authors declare that they have no competing interests.

## Authors' contributions

RL conducted the qualitative data analysis, contributed to study design and data collection, and wrote the first draft of the manuscript. LK and MH contributed to study design and data analysis. GPD and AW contributed to study design, while REM contributed to study design and data collection. All authors read, contributed to, and approved the final manuscript.

## Supplementary Material

Additional file 1 **The role of the community health providers involved in the project**. Description of the role of various community health providers involved in the project. Click here for file
