# Supplementary material for: An exploration of how clinician attitudes and beliefs influence the implementation of lifestyle risk factor management in primary healthcare: a grounded theory study
Source: Implement Sci. 2009 Oct 13;4:66. doi: 10.1186/1748-5908-4-66 (PMC2770564; doi:10.1186/1748-5908-4-66)
Supplement: Additional file 1 — The role of the community health providers involved in the project. Description of the role of various community health providers involved in the project. [file 1748-5908-4-66-S1.DOC]

**Additional File 1: The role of the community health providers involved in the project**

Below is a description of the role of various community health providers involved in the project.

**Registered and enrolled generalist community nurses:**

In Australia, registered nurses undertake a three-year tertiary education program. Enrolled nurses undertake training from one to two years at a technical college receiving a certificate or diploma depending on the state. Enrolled nurses work with registered nurses to provide patients with basic nursing care. Within the project, registered nurses undertook the initial patient assessment and care planning, and enrolled nurses assisted with the implementation of the care plan. In team one, generalist community nursing predominately conducted home visits and saw patients for wound management, medication administration, chronic disease management, and palliative care. The majority of patients were referred following discharge from hospital and were over 65 years of age. In team two, each of the ‘generalist’ community nurses had a specific role such as diabetes education, cardiac rehabilitation, women’s health, palliative care, disabilities, and geriatric care. Most patients were seen on an individual basis, either through home visits or at clinics held at the community health centre.

**Primary healthcare (PHC) nurses:**

PHC nurses in team three were all registered nurses. Each PHC nurse worked with a number of rural or remote communities providing regular ‘drop in’ clinics focusing on health screening and monitoring that were held at community venues, such as the local community hall or church. PHC nurses in this team also reported spending at least one-half their time conducting community education and development activities, such as undertaking needs assessment of local communities, providing group education programs for adults, health education programs in schools, and health screening at community venues or events.

**Child and family nurses:**

Child and family health nurses are registered nurses or midwives (often with graduate certificate or graduate diploma level qualifications in maternal, child, and family health) who provide a range of services to families with infants and young children. This includes health education and promotion in the form of baby clinics, parenting groups, individual patient care, and primary school involvement. Services may vary from state to state and territory, but the goals are similar: to provide infants, children, and adolescents and their families with a range of professional services that have a strong emphasis on prevention, early intervention, support, and referral to other services.

**Aboriginal health worker (AHW):**

AHWs provide clinical and primary healthcare for individuals, families, and community groups. This includes health screening, monitoring, and community development work, as well as liaising between the Aboriginal community and other health professionals. Most AHWs undertake a certificate III or IV in Aboriginal Primary Healthcare Work at a technical college, however educational requirements vary nationally. In this project, AHWs in team three worked along side PHC nurses in a number of rural and remote communities providing a combination of health screening, monitoring, and undertaking community development activities.

**Allied health practitioners:**

Allied health practitioners working in team two included a social worker, psychologist, speech pathologist, occupational therapist, and dietitian. Team three had two allied health practitioners: a social worker and counsellor.
